# Maximizing electrical output and reducing heat-related losses in photovoltaic thermal systems with a thorough examination of flow channel integration and nanofluid cooling

**DOI:** 10.1038/s41598-023-44272-7

**Published:** 2023-10-08

**Authors:** F. M. Allehiany, Abid A. Memon, M. Asif Memon, Amsalu Fenta

**Affiliations:** 1 Department of Mathematical Sciences, College of Applied Sciences, Umm Al-Qura University, Makkah, Saudi Arabia; 2 Department of Mathematics and Social Sciences, Sukkur IBA University, Sukkur, 65200 Pakistan; 3 Department of Mathematics and Statistics, Faculty of Applied Sciences and Technology, Universiti Tun Hussein Onn, Malaysia, 86400 Batu Pahat, Johar Malaysia; 4 Department of Physics, Mizan Tepi University, PO Box 121, Tepi, Ethiopia

**Keywords:** Engineering, Materials science, Mathematics and computing

## Abstract

In recent years, global energy demand has surged, emphasizing the need for nations to enhance energy resources. The photovoltaic thermal (PV/T) system, capable of generating electrical energy from sunlight, is a promising renewable energy solution. However, it faces the challenge of overheating, which reduces efficiency. To address this, we introduce a flow channel within the PV/T system, allowing coolant circulation to improve electrical efficiency. Within this study, we explore into the workings of a PV/T system configuration, featuring a polycrystalline silicon panel atop a copper absorber panel. This innovative setup incorporates a rectangular flow channel, enhanced with a centrally positioned rotating circular cylinder, designed to augment flow velocity. This arrangement presents a forced convection scenario, where heat transfer primarily occurs through conduction in the uppermost two layers, while the flow channel beneath experiences forced convection. To capture this complex phenomenon, we accurately address the two-dimensional Navier–Stokes and energy equations, employing simulations conducted via COMSOL 6.0 software, renowned for its utilization of the finite element method. To optimize heat dissipation and efficiency, we introduce a hybrid nanofluid comprised of titanium oxide and silver nanoparticles dispersed in water, circulating through the flow channel. Various critical parameters come under scrutiny, including the Reynolds number, explored across the range of 100–1000, the volume fractions of both nanoparticle types, systematically tested within the range of 0.001–0.05, and the controlled speed of the circular cylinder, maintained within the range of 0.1–0.25 m/s. It was found that incorporating silver nanoparticles as a suspended component is more effective in enhancing PV/T efficiency than the addition of titanium oxide. Additionally, maintaining the volume fraction of titanium oxide between 4 and 5% yields improved efficiency, provided that the cylinder rotates at a higher speed. It was observed that cell efficiency can be regulated by adjusting four parameters, such as the Reynolds number, cylinder rotation speed, and the volume fraction of both nanoparticles.

## Introduction

The PV/T system, a cutting-edge technology for harnessing electrical energy from sunlight, is a subject of widespread global research and study. Yet, a known challenge in its operation is the reduction in efficiency as atmospheric temperatures rise^1,2^. To address this issue and improve the performance of PV/T systems, a range of strategies has been explored. One such approach involves the introduction of a coolant, such as nanofluids, into the system's flow channel, or the incorporation of a rotating device within the flow. Nanofluids, characterized by the infusion of metallic particles into a base fluid like water or glycol, offer enhanced thermal properties when compared to the base fluid in isolation^3,4^. Meanwhile, the integration of a rotating device in the flow channel serves to induce mixing and turbulence, thereby facilitating more effective heat transfer during the convection process. These methodologies have consistently demonstrated their potential to enhance PV/T system efficiency. However, it's worth noting that a comprehensive exploration of their effects is still an area where in-depth research is necessary.

Several studies have delved into the utilization of nanofluids in the context of PV/T systems. For instance, Tiwari and Sodha^5^ explored an integrated PV/T system, investigating four distinct configurations of PV/T and employing both water and air as coolants. Their findings suggested that, with the exception of one configuration, the use of water as a coolant yielded advantages across all configurations within the PV/T system. Notably, they observed that the system's efficiency reached 65% during the summer and 77% in the winter. In a related study, Tiwari et al.^6^ focused on a solar-based PV/T system while keeping the flow rate and collection temperature constant. Their investigation unveiled a direct correlation between the system's water temperature and the local climate. Increasing the flow rate emerged as a favorable factor for enhancing PV/T efficiency. Conversely, elevating the collection temperature was found to decrease efficiency. Furthermore, they conducted an exergy analysis, revealing that the system's efficiency peaked at a specific flow rate. In the final phase of their research, they meticulously evaluated the electrical power generated by the system.

Tonui and Tripanagnostopoulos^7^ conducted a detailed investigation into the influence of temperature on the efficiency of a photovoltaic thermal (PVT) system, proposing the implementation of an air-cooling solution. Nevertheless, the air-cooling method presented inherent limitations due to the relatively low density and thermal conductivity of air. This necessitated an enhancement in the heat transfer rate. In response, they recommended the incorporation of metallic sheets and fins, coupled with an air duct, which substantially augmented heat transfer capabilities. The adapted system was rigorously compared to a conventional air-cooled system, revealing that this modification proved effective across a range of temperatures. In a different approach, Barone et al.^8^ delved into a technique designed to elevate the efficiency of a PV system. They developed an innovative and cost-effective prototype PV/T system intended to deliver both electricity and hot water to a building. This system underwent testing in three distinct climate zones in Europe, and simulation results further corroborated its effectiveness. In addition to the performance assessment, the study offered valuable insights by presenting various design options. These design alternatives were formulated following meticulous analyses encompassing energy considerations and economic sensitivity evaluations.

Bianco et al.^9^ embarked on an endeavor to harness nanofluids in the pursuit of an efficient PV/T system that could yield maximum electrical energy output. To this end, they ingeniously employed a nanofluid composite, composed of Al_2_O_3_ and water, as the flowing medium within the PV/T flow channel. Their meticulous numerical modeling affirmed the remarkable impact of nanofluid-based cooling on elevating the system's efficiency. Notably, their investigation shed light on the crucial observation of pressure drop within this context. Furthermore, they illuminated a pivotal insight: when the reduction in thermal entropy generation outpaces the decrease in friction entropy generation, it leads to a notable enhancement in the system's efficiency. In a separate pursuit, Chow et al.^10^ undertook an in-depth analysis of a flat plate solar collector, with the aim of optimizing its efficiency. The primary question that guided this study was whether introducing a glass cover would enhance the thermal performance of a solar collector. To address this question, the researchers meticulously examined six critical factors. Their comprehensive findings revealed that the inclusion of a glass cover significantly bolstered both the thermal and energy output, as discerned through a thorough analysis based on the first law of thermodynamics. Additionally, the study discerned that unglazed pipes proved more advantageous when the goal was to augment cell efficiency, water mass, and wind velocity. Conversely, the use of glazed pipes was found to be more effective when operating in conditions of elevated ambient temperatures.

Ebrahimnia-Bajestan^11^ conducted a comprehensive exploration into the remarkable thermal properties of nanofluids, particularly their utility in solar heat exchangers. This multifaceted study employed a combination of numerical modeling and experimental investigations. Within this framework, nanofluids, comprising TiO_2_ and water, were employed as the flowing medium in a heated pipe. The research unveiled the impressive heat transfer enhancements facilitated by TiO_2_ when employed in conjunction with water. Specifically, a substantial 21% boost in heat transfer rate was observed. The study culminated in the development of a modified and efficient model, forged through rigorous comparisons of different models. This modified model skillfully delineated how factors such as particle concentration, diameter, and choice of flowing material could synergistically enhance heat transfer. These findings hold great promise for their application in solar collector systems. Gorji and Ranjbar^12^ undertook a numerical investigation centered around nanofluid-based solar collectors. Their research delved into the thermal attributes of three distinct nanoparticle types, with water serving as the base fluid. The results were meticulously scrutinized using response surface methods. The study revealed the pivotal role of silver and graphite nanofluids in augmenting thermal energy and exergy performance, particularly when synergistically combined with magnetite. The research also offered valuable insights by suggesting specific operational conditions for optimizing this application.

Herrando et al.^13^ presented a captivating array of 26 alternative designs for hybrid solar collectors, systematically benchmarking them against a commercial reference case. These proposed designs introduced a contemporary flat-box structure crafted from alternative materials, effectively reducing weight and cost while simultaneously maintaining or even enhancing overall performance. The study scrutinized these designs through a sophisticated computational model, ultimately singling out a polycarbonate flat-box design featuring rectangular channels as a highly promising alternative to commercial collectors. This alternative design exhibited improved thermal performance, reduced weight, and diminished investment costs. Furthermore, the study underscored the lower stress levels exhibited by the proposed flat-box design, primarily attributed to the larger thermal expansion of the absorber-exchanger. In a comprehensive review, Jia et al.^14^ set forth a compelling objective: to enhance the energy efficiency of solar cells by advancing the development of hybrid photovoltaic and thermal collector systems. These innovative systems aim to concurrently generate electrical power and harvest thermal energy, thereby elevating the overall system efficiency. The review's exhaustive examination spanned various types of PV/T systems, the selection of working fluids, and consideration of diverse environmental conditions. The authors contended that the creation of novel PV/T systems necessitates an intensified focus on precise modeling, exploration of innovative materials, and the strategic design of an integrated energy storage system.

Jia et al.^15^ conducted a comprehensive analysis to investigate the multifaceted influence of nanofluid type, volume concentration, and PV collector parameters on the performance of PV/T collectors. Their insightful research revealed that employing Al2O3/water nanofluid, coupled with a higher mass flow rate of the nanofluid, yielded notably improved electrical and thermal power outputs. Furthermore, the study illuminated that diminishing the channel height resulted in an augmented removal of heat and an upswing in the thermal power generated by the PV/T collector.

In a complementary study, Mahian et al.^16^ embarked on an extensive exploration of the consequences of nanofluid flow on heat transfer dynamics and entropy generation within a flat plate solar collector. This intricate analysis encompassed critical variables such as nanoparticle size, volume concentration, tube roughness, and thermophysical models. The research unveiled that elevating the nanofluid volume fraction was associated with an increase in outlet temperature, while the Nusselt number exhibited a converse relationship. Intriguingly, the study also brought to light that entropy generation diminished with increasing nanofluid concentration, yet ascended with the introduction of tube roughness. The paper concluded by pinpointing a critical mass flow rate at which the impact of roughness on entropy generation became notably influential.

Nahar et al.^17^ have directed their focus towards the implementation of hybrid photovoltaic-thermal (PV/T) systems, seeking to provide sustainable energy solutions. Their study introduces an innovative thermal collector design that eliminates the need for an absorber plate, thereby enhancing heat transfer. The system's performance was meticulously evaluated through a combination of numerical simulations and practical experiments. Notably, the research revealed that the thermal performance of the PV/T system, even in the absence of an absorber plate, closely rivaled that of traditional systems. This pioneering design holds great potential for influencing the configurations of future thermal collectors. In a parallel endeavor, Nasrin et al.^18^ embarked on a quest to enhance the thermal performance of PV modules by incorporating a novel thermal collector design within a PV/T system. They employed a water/MWCNT nanofluid to bolster thermal efficiency, while actively maintaining cooling through the use of a centrifugal pump and radiator. The study encompassed 3D numerical simulations and indoor experiments conducted under varying conditions, all of which yielded highly encouraging outcomes. Notably, water cooling resulted in a significant 9.2% boost in PV performance, while the utilization of nanofluids contributed to approximately 4% and 3.67% enhancements in thermal performance in numerical and experimental investigations, respectively. This research ultimately showcased the superior efficiency of the PVT system employing nanofluids when compared to a water-cooled system.

Pang et al.^19^ delved into the intricate interplay between operating temperature and the efficiency of flat plate photovoltaic/thermal (PV/T) collectors. Their investigation categorized four distinct configurations based on the choice of operating media (air, water, nanofluids, and bi-fluids) and meticulously evaluated their respective advantages. Remarkably, water-type PV/T collectors exhibited superior efficiency, owing to the elevated specific heat capacity of water, while nanofluid-type PV/T collectors emerged as the frontrunners in overall efficiency, attributable to the remarkable thermal conductivity of nanoparticles dispersed within a base fluid. The study additionally considered the impact of absorber structure, glass cover, and geographical location on overall system performance. Prakash^20^ undertook a comprehensive theoretical exploration of a hybrid solar system, ingeniously combining photovoltaic electricity generation with thermal energy collection. Their pioneering design features a duct beneath the solar panel, facilitating heat extraction to enhance the efficiency of the solar cells. A mathematical model grounded in energy balance equations was meticulously developed to forecast the system's performance over time. A thorough comparative analysis with traditional photovoltaic panels underscored the remarkable effectiveness of the hybrid system.

A PV/T system with a similar configuration, featuring a deep cavity as the flow channel, was examined in a previous study by Memon et al.^21^. This investigation assessed the passage of Cu and Alumina nanofluids, aiming to lower the cell temperature and enhance electrical efficiency. The simulation was conducted using COMSOL Multiphysics, and the parameters used to evaluate efficiency effects included the Reynolds number, aspect ratio (total height to the height of the deep cavity attached to the flow channel), volume fraction, and the inlet temperature of the nanofluids. The results indicated that cell efficiency could be optimized to 6% when the volume fraction of copper was set at 10%. Furthermore, it was observed that, when keeping Re = 1000 constant, increasing the volume fraction of nanofluids improved efficiency by 0.3%. In a recent research article, a novel approach was explored by Memon^22^ to boost the electrical efficiency of a photovoltaic thermal (PV/T) system with a backward step flow channel. The researchers introduced a hybrid nanofluid comprising copper and aluminum oxide as the base, with water as the carrier fluid. The objective was to harness the synergistic benefits of these nanoparticles for enhanced energy generation. To comprehensively investigate this phenomenon, state-of-the-art simulation software, COMSOL Multiphysics, was employed. The study delved into a range of crucial parameters, including the Reynolds number, volume fraction of nanoparticles, and the aspect ratio (inlet to outlet height), to evaluate their impact on system efficiency.

A photovoltaic thermal system was tested by Alghamdi^23^ using a trapezoidal flow channel, integrating copper and polycrystalline silicon, and a mixture of titanium oxide and silver nanomaterials in water. Various parameters were examined, including volume fraction, Reynolds number, and aspect ratio. The study found that increasing Reynolds number from 100 to 1000 reduced cell temperature by 50%, boosting efficiency. Higher inlet height was also suggested to enhance system performance by allowing more fluid into the system. These results promise improved photovoltaic thermal system efficiency. Another commendable work was conducted by Aglawe et al.^24^, focusing on the design and simulation of a microchannel heat sink device integrated with a heat-producing device. The study included a parametric evaluation considering heat flux, mass flow, inlet temperature, and nanofluid concentration. It was established that the heat sink efficiently reduced the device's temperature. When utilizing a 0.3% volume fraction and a flow rate of 8 kg/s, the maximum heat transfer rate reached 13,693 W/m^2^ K at an inlet temperature of 35 °C. In an investigation conducted by Yousefi et al.^25^, a system with an external resistance was assessed, incorporating phase change material with copper foams. This configuration (case 3) demonstrated faster thermal diffusion, reducing overall thermal resistance and enhancing heat flux through the phase change material. Case 3 effectively reduced hot and cold side temperatures by up to 8% and 7%, respectively, without compromising output voltage. This solution proves practical for autonomous systems, offering zero-cooling and improved safety when exposed to dynamic thermal sources.

Having conducted a comprehensive review of existing research, it has become evident that a substantial increase in the demand for electrical energy is looming on the horizon. To meet this impending energy deficit, it is imperative to harness the potential of the sun, a renewable and abundantly available energy source, for electricity generation. Consequently, there is a compelling need to delve into the photovoltaic thermal system and enhance its efficiency. With this objective as our guiding principle, this article centers on the investigation of the PV/T system. Our approach revolves around the implementation of a streamlined PV/T system design complemented by the integration of nanofluid-based cooling. Additionally, we have introduced a dynamic cylinder within the flow channel to fortify the electrical efficiency of the PV/T system. In our pursuit of elevating cell efficiency, we embark on a comprehensive analysis of pivotal factors, including the Reynolds number, the volume fraction of nanofluids, and the speed of the moving cylinder. Our study culminates with the presentation of a set of discerning recommendations and practical suggestions.

## Problem formulation

**Figure 1:**
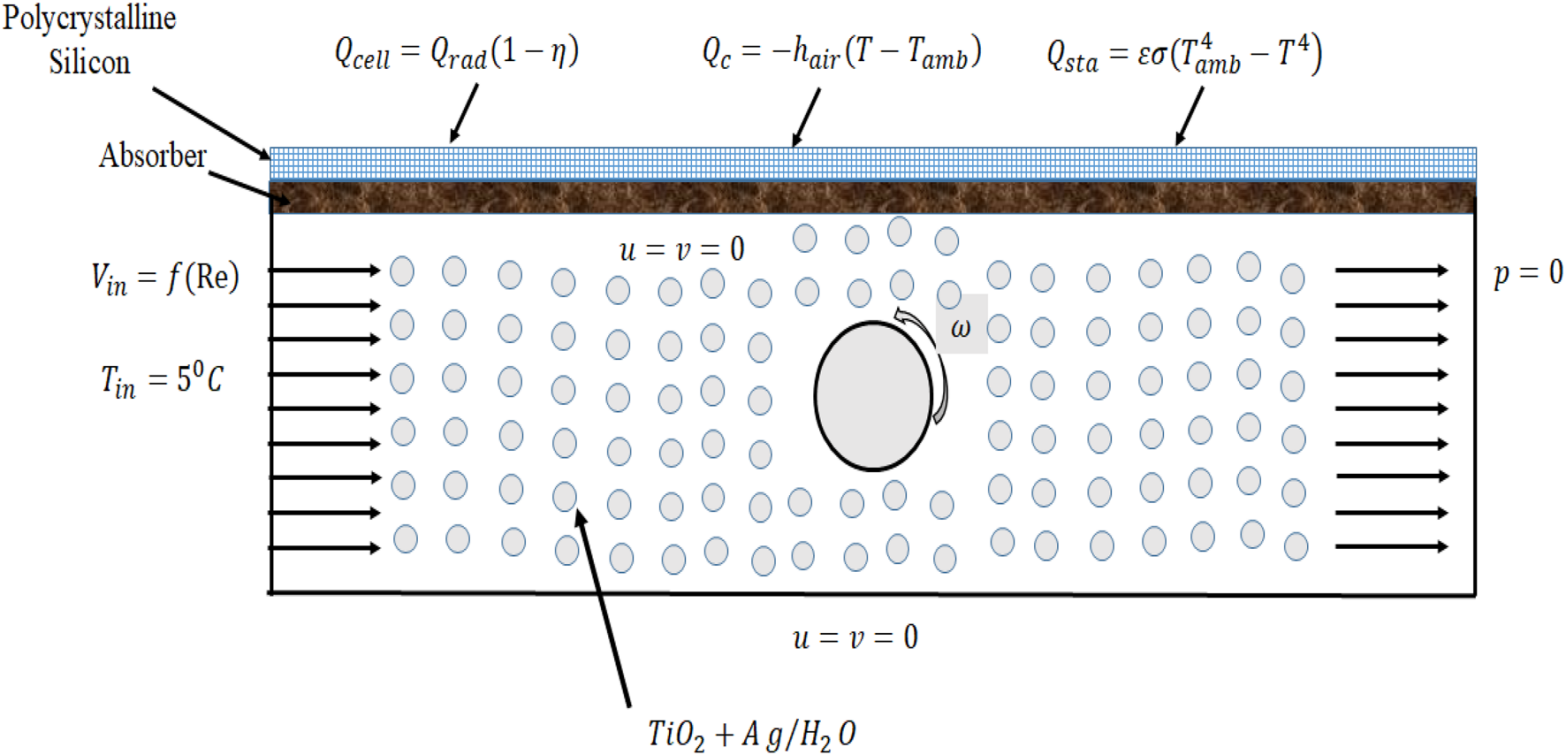
Creating a Photovoltaic Thermal System (PV/T) and defining boundary conditions for finite element analysis.

Typically, problems involving the evaluation of the electrical efficiency of photovoltaic thermal systems are approached in three dimensions. However, if the channel exhibits symmetry along the z-axis, a two-dimensional coordinate system can be effectively employed, simplifying the analysis process and saving valuable time. As depicted in Fig. 1, our PV/T system features a straightforward configuration, consisting of three distinct domains. The uppermost domain is crafted from polycrystalline silicon, a prevalent choice for solar energy production. Subsequent to this, we have the copper absorber domain. To enhance the system's efficiency, we've integrated a flow channel equipped with a centrally placed rotating cylinder. This cylinder spins in an anticlockwise direction at a velocity represented as $\omega$, and its radius-to-total height ratio is marked as 'a'. For practicality, we've imposed three distinct flux conditions on the silicon surface, thoughtfully illustrated in Fig. 1. It's crucial to recognize that, in reality, only about 20% of the sun's radiant energy is converted into electricity, with the remainder being transformed into thermal energy. The boundary conditions for these scenarios are eloquently expressed through Eqs. (11)–(14). Comprehensive details of the parameters considered for channel measurement are meticulously documented in Table 1.

**Table 1:** The parameters used to create the flow channel in the PV/T system.

Symbols	Values	Description
$h_{in}$	20 (mm)	Height of inlet of the flow channel
$h_{out}$	$h_{in}$	Height of outlet of the flow channel
$L_{{t,}_{cell}}$	100 (mm)	Total length of the flow channel
$H_{{t,}_{abs}}$	1 (mm)	Height of the absorber made of copper
$H_{{t,}_{Si}}$	1 (mm)	Height of the silicon cell
A	$L_{cell} H_{in} - \pi R^{2}$ (mm^2^)	Total area of the flow channel
P	$2L_{cell} + 2H_{in} + 2\pi R$ (mm)	Perimeter
$d_{h}$	4*A/P (mm)	Hydraulic diameter
Ar	0.25	Aspect ratio
R	a $H_{in}$ (mm)	Radius of the cylinder
$\omega$	0.01, 0.05, 0.09, 0.1, 0.2	Velocity of cylinder

The uppermost domains within the PV/T system, directly exposed to the sun's radiant energy and featuring the silicon component as the primary material, are primarily characterized by pure conduction mechanisms. Conversely, within the flow channel, a forced convection phenomenon takes place. The analysis of pure conduction in these upper domains is facilitated using two-dimensional equations. The transmission of heat energy commences with the absorption of sunlight by the silicon panel, subsequently transferring this thermal energy to the absorber material. To comprehensively investigate this heat transfer process occurring within the silicon and copper components, we'll rely on the thermo-physical properties, thoughtfully detailed in Table 2.

**Table 2:** Material properties of silicon and copper to construct the top two layers of PV/T system^26^.

Symbols	Values	Description
$(c_{p} )_{Cu}$	386 (J/(kg*K))	Specific heat of copper at constant pressure
$\rho_{sili}$	2330 (kg/m^3^)	Density of silicon
$(c_{p} )_{sili}$	716 (J/(kg*K))	Heat capacity of silicon
$(\kappa_{t} )_{sili}$	157 (W/(m*K))	Thermal conductivity of silicon
$\beta$	0.003 (1/K)	Temperature coefficient
$\rho_{Cu}$	8900 (kg/m^3^)	Density of copper
$(\kappa_{t} )_{Cu}$	398 (W/(m*K))	Thermal conductivity of copper
$\varepsilon_{sili}$	0.9	Emissivity
$\nu_{ref}$	0.2	Reference efficiency of cell
$Ac$	$Lt_{cell} Ht_{silicon}$	Area of one cell

In our pursuit to mitigate the temperature within the PV/T system, we employ a hybrid coolant mixture. This innovative solution combines two nanoparticles meticulously blended with water. As the hybrid mixture enters the channel, it possesses an initial x-velocity $u_{1} = V_{in}$ at the entrance and maintains a specific starting temperature, T_in_, as outlined in the accompanying table. Our focus within the flow channel revolves around the phenomenon of forced convection. This exploration necessitates the presence of two distinct nanoparticle types: silver and titanium oxide. Notably, silver outshines titanium oxide in terms of thermal conductivity, making it a promising choice for our cooling application. The detailed thermal properties of these nanoparticles, along with those of the water-based mixture, can be found in Table 3, providing a solid foundation for our experimental endeavors. Additionally, we introduce a standard solar radiation intensity of Qrad = 1000 W/m^2^, a crucial parameter for our comprehensive analysis.

**Table 3:** Properties of nanofluids to carry the convection process^27^.

Symbols	Description	Values
$\rho_{np1}$	Density of $TiO_{2}$	4250 (kg/m^3^)
$\rho_{np2}$	Density of (Ag)	10.5 (kg/m^3^)
$\rho_{np}$	Total density of nanoparticles	$\frac{{\phi_{1} \rho_{np1} + \phi_{2} \rho_{np2} }}{{\phi_{1} + \phi_{2} }}$
$Cp_{np1}$	Specific heat at constant pressure of Titanium oxide	686.2 (J/(kg K))
$Cp_{np2}$	Specific heat at constant pressure of silver	235 (J/(kg K))
$Cp_{np}$	Over all specific heat at constant pressure of nanoparticles	$\frac{{\phi_{1} \rho_{np1} Cp_{np1} + \phi_{1} \rho_{np1} Cp_{np1} }}{{\rho_{np} \phi }}$
$\phi_{2}$	Volume fraction of (Ag)	0.001, 0.01, 0.04, 0.05
$\phi_{1}$	Volume fraction of $TiO_{2}$	0.001, 0.01, 0.04, 0.05
$\phi$	Total volume fraction of nanoparticles in the base fluid	$\phi_{1} + \phi_{2}$
$\kappa_{np1}$	Thermal conductivity of $TiO_{2}$	8.952 (W/(mK))
$\kappa_{np2}$	Thermal cond. Of silver (Ag)	429 (W/(mK))
$\kappa_{np}$	Overall thermal conductivity	$\frac{{\phi_{1} \kappa_{np1} + \phi_{2} \kappa_{np2} }}{\phi }$
$\rho_{bf}$	Density of water	998 (kg/m^3^)
$\rho_{hnf}$	Total density of nanofluids	$\rho_{bf} (1 - \phi ) + \phi \rho_{np}$
$Cp_{bf}$	Specific heat at constant pressure of water	4182 (J/(kg K))
$Cp_{hnf}$	Total specific heat of the nanofluids at the constant pressure	$\frac{{\rho_{bf} (1 - \phi )Cp_{bf} + \rho_{np} (1 - \phi )Cp_{np} }}{\phi }$
$\kappa_{bf}$	Thermal conductivity of water	0.597 (W/(mK))
$\kappa_{hnf}$	Total thermal conductivity of hybrid mixture	$\left( {\frac{{\kappa_{np} + 2\kappa_{bf} + 2(\kappa_{np} - \kappa_{bf} )\phi }}{{\kappa_{np} + 2\kappa_{bf} - 2(\kappa_{np} - \kappa_{bf} )\phi }}} \right)\kappa_{bf}$
$\mu_{bf}$	Water viscosity	0.000998 (Pas)
$\mu_{hnf}$	Viscosity of nanofluid	$\mu_{bf} \frac{1}{{(1 - \phi )^{2.5} }}$
Re	Reynolds number	100, 400, 700, 1000
$u_{in}$	Inlet velocity	${\text{Re}} \frac{{\mu_{hnf} }}{{\rho_{hnf} D_{h} }}$
T_in_	Inlet temperature	5^0^C
Q_rad_	Heat flux	1000 (W/m^2^)
T_ref_	Reference Temperature	25 °C
${\text{T}}_{{{\text{amb}}}}$	Ambient Temperature	45 °C

Where, np, nanoparticle; nf = nanofluids; hnf,  hybrid nanofluids; bf,  basefluid.

### Partial differential equations and comsol working

We have connected the power of the incompressible Navier–Stokes equation and the energy equation as our tools for meticulously observing this phenomenon. These equations are skillfully guided by the boundary conditions thoughtfully presented in Fig. 1, and we eagerly anticipate unveiling our findings. In our pursuit of accuracy, we recognize the paramount importance of employing a robust numerical scheme. In our estimation, the finite element method stands as the ideal choice. To this end, we have leveraged the capabilities of COMSOL Multiphysics 6.0, a finite element-based software built on Python programming, renowned for its adeptness at simulating complex phenomena. For our analysis, we have maintained a steady-state configuration, signifying its time-independent nature. Mathematically, we've encapsulated the entire phenomenon within Eqs. (1)–(4). Specifically, Eqs. (1)–(3) are tailored to the flow channel, where we leverage the thermo-physical properties of nanofluids to extract numerical solutions for fluid behavior. In parallel, Eq. (4), a two-dimensional heat equation, transcends domain boundaries, facilitating the simulation of heat exchange across all three domains. Its functionality is intricately intertwined with the thermal properties of the domains where heat flow dynamically unfolds.

1 $$\frac{{\partial u_{1} }}{\partial x} = - \frac{{\partial u_{2} }}{\partial y}$$

2 $$u_{1} \frac{{\partial u_{1} }}{\partial x} + u_{2} \frac{{\partial u_{1} }}{\partial y} + \frac{1}{{\rho_{hnf} }}\frac{\partial p}{{\partial x}} = \mu_{hnf} \left( {\frac{{\partial^{2} u_{1} }}{{\partial x^{2} }} + \frac{{\partial^{2} u_{1} }}{{\partial y^{2} }}} \right)$$

3 $$u_{1} \frac{{\partial u_{2} }}{\partial x} + u_{1} \frac{{\partial u_{2} }}{\partial y} + \frac{1}{{\rho_{hnf} }}\frac{\partial p}{{\partial y}} = \mu_{hnf} \left( {\frac{{\partial^{2} u_{2} }}{{\partial x^{2} }} + \frac{{\partial^{2} u_{2} }}{{\partial y^{2} }}} \right)$$

4 $$u_{1} \frac{\partial T}{{\partial x}} + u_{2} \frac{\partial T}{{\partial y}} = \alpha_{hnf} \left( {\frac{{\partial^{2} T}}{{\partial x^{2} }} + \frac{{\partial^{2} T}}{{\partial y^{2} }}} \right)$$

### Other computational formula

Here, we provide several key parameters for post-processing. Equation (5) outlines the formula for the average Nusselt number, which incorporates the heat transfer coefficient described in Eq. (6). Equation (6) presents the formula for the bulk temperature, denoted as Tb, necessary for calculating its value using Eq. (7). In Eqs. (8) and (9), we encounter the Prandtl number and cell efficiency, respectively. Equation (10) offers a correlation for the Reynolds number, which serves as a valuable tool for verification purposes. To complete this set of equations, we've defined three fundamental conditions for heat fluxes imposed on the silicon domain. Equation (11) quantifies the total heat flux directed toward the panel. It's worth noting that a portion of the incident sunlight is converted into thermal energy, as indicated in Eq. (12). Additionally, Eq. (13) characterizes the heat reflection occurring in the panel's immediate surroundings. Finally, Eq. (14) elucidates the heat transfer occurring through the ambient temperature enveloping the panel.

5 $${\text{Average Nusselt Number:}}\quad Nu_{avg} = \frac{{D_{h} h}}{{\kappa_{hnf} }}$$

6 $${\text{Heat Transfer coefficient:}}\quad h = \frac{{T_{x} k_{hnf} }}{{T_{b} - T}}$$

7 $${\text{Bulk Temperature:}}\quad T_{b} = \frac{{\int\limits_{0}^{{H_{t} }} {\frac{{\partial u_{1} }}{\partial x}Tdx} }}{{\int\limits_{0}^{{H_{t} }} {\frac{{\partial u_{1} }}{\partial x}dx} }}$$

8 $${\text{Prandtl Number:}}\quad \Pr = \frac{{Cp_{hnf} \mu_{hnf} }}{{\kappa_{hnf} }}$$

9 $${\text{Cell efficiency}}\quad \nu_{cell} = \nu_{ref} [1 - \beta (T_{cell} - T_{ref} )]$$

10 $${\text{Nusselt Number by Pak and Cho }}\left[ {{28}} \right]\quad Nu_{P\& C} = 0.021{\text{Re}}^{0.8}{\Pr}^{0.5}$$

11 $$Q_{abs} = Q_{cell} - (Q_{str} + Q_{c} )$$

Here,

12 $${\text{The heat source in the domain:}}\quad Q_{cell} = Q_{rad} (1 - \eta )$$

13 $${\text{Heat reflected surrounding:}}\quad Q_{rad} = \varepsilon \sigma (T_{cell}^{4} - T_{amb}^{4} )$$

14 $${\text{Heat transferred to the PV - panel by surroundings:}}\quad Q_{c} = h(T_{amb}^{4} - T^{4} )$$

## Mesh independent study and validation of simulation

One of the notable advantages of employing numerical procedures lies in their capacity to break down complex systems into smaller, manageable segments and subsequently employ specific methodologies to derive solutions. The system and its corresponding domain can be subdivided into numerous segments, but it's important to bear in mind that the solution obtained is always an approximation. In the realm of numerical analysis, the concept of accuracy level comes to the forefront, and it depends on how finely we partition the domain. This process of dividing the complete domain into smaller subdomains is often referred to as the meshing procedure. It's worth noting that the accuracy of any numerical solution continually improves with an increased mesh density or a higher number of elements.

**Figure 2:**
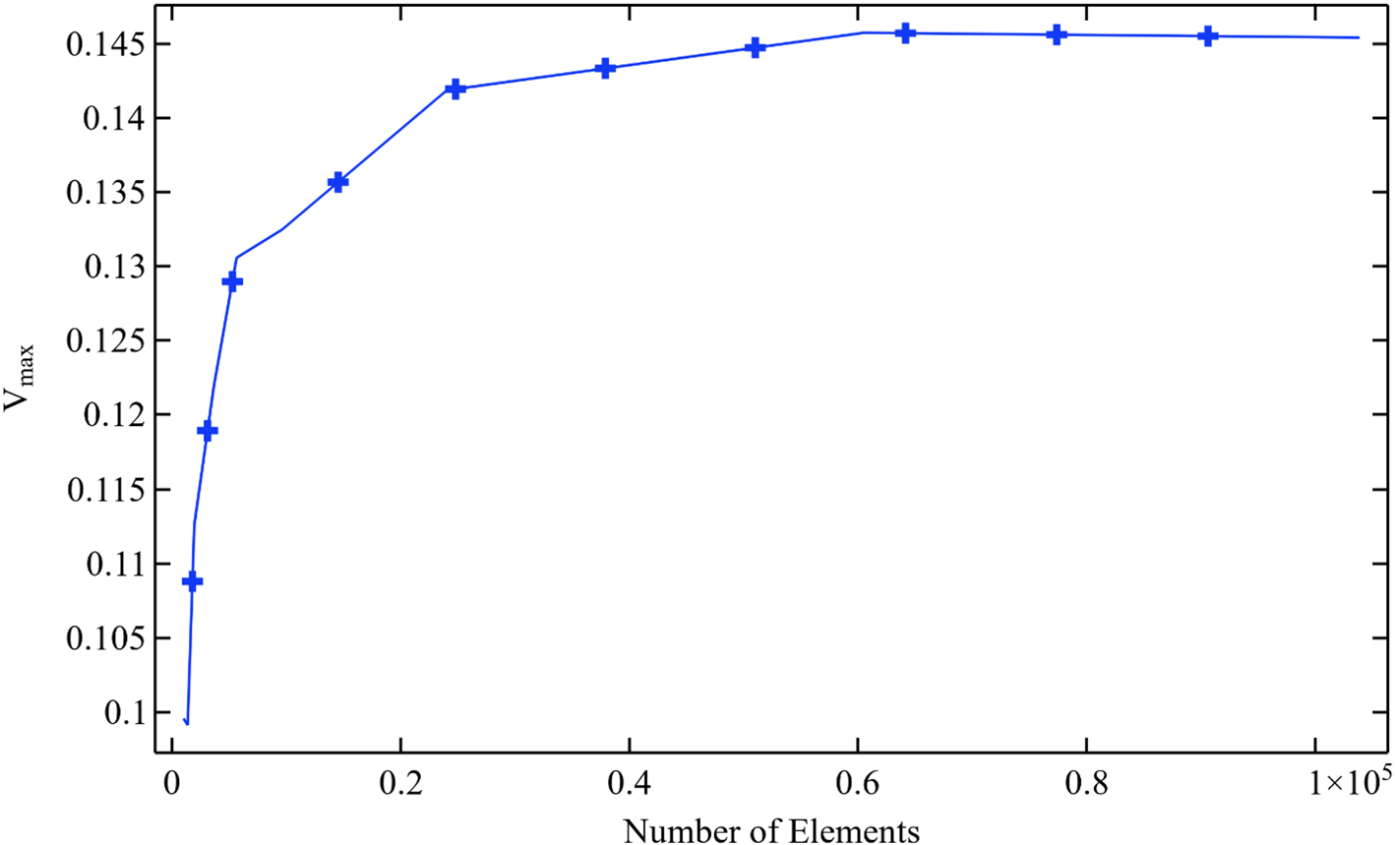
Validity of numerical solution through the mesh independent test when Re = 1000, $\phi_{1}$ = $\phi_{2}$ = 0.5 and $\omega$ = 0.1 m/s.

To elevate the accuracy level of our numerical solution, we have conducted a mesh-independent test. In this mesh-independent study, we calculated the maximum velocity at the outlet of a flow channel while progressively increasing the number of elements from 1000 to 100,000. As depicted in Fig. 2, the numerical solution demonstrates clear improvement as the number of domain partitions grows. Figure 2 clearly indicates that the numerical solution reaches a state of mesh-independence at around 60,000 elements. To further enhance the reliability, practicality, and utility of our numerical solution, we have conducted this computation with 100,000 elements.

**Figure 3:**
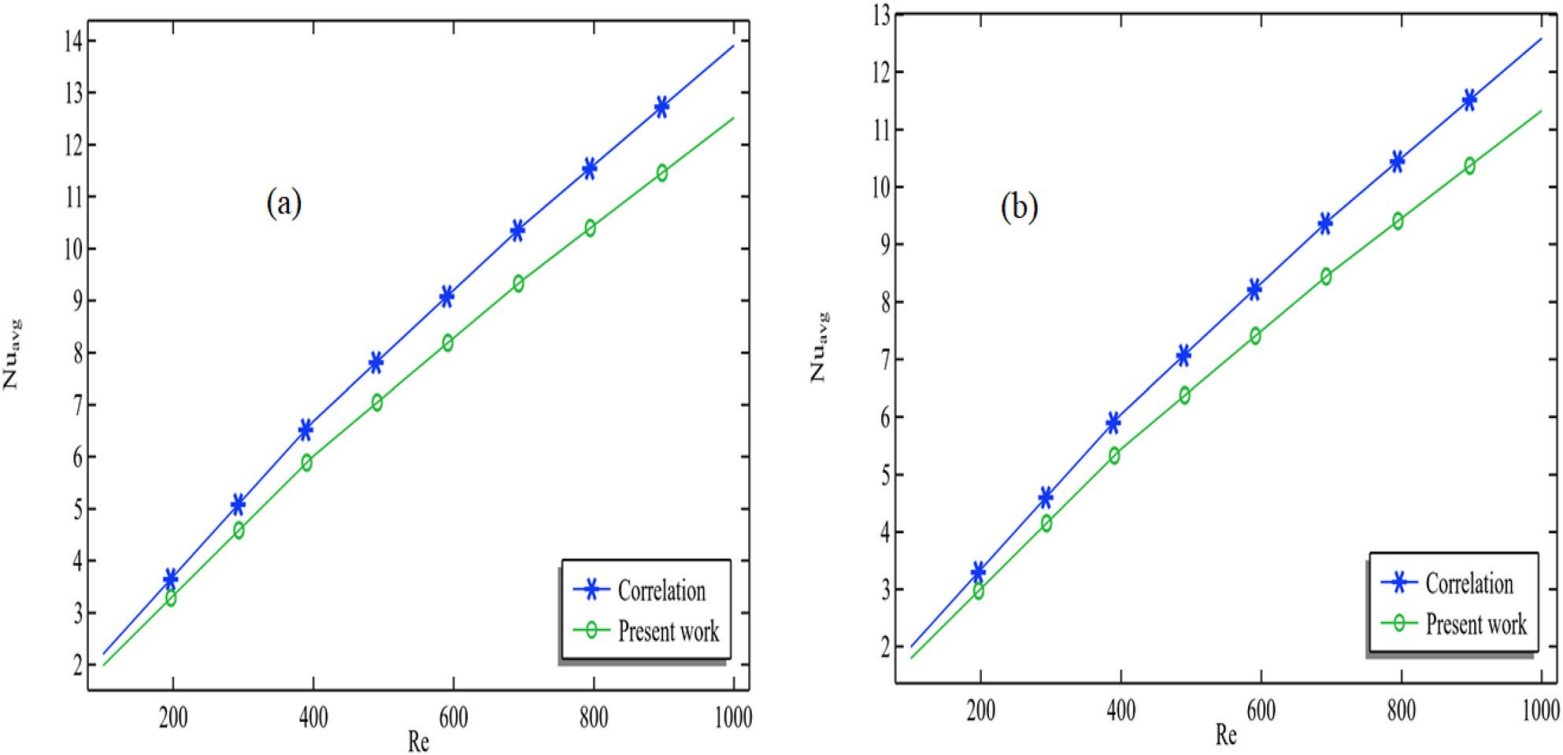
Validation of the average Nusselt number with Pak and Cho correlation^28^ when $\phi_{2}$ = 0.001, $\omega$ = 0.01 m/s (**a**) $\phi_{1}$ = 0.001 and (**b**) $\phi_{1}$ = 0.05.

In Fig. 3, we conducted a comparative analysis between the numerical solution of the average Nusselt number obtained from our present simulation and the correlations presented by Pak and Cho^23^. Our findings reveal a close match between the Nusselt number derived from our numerical solution and the experimental correlation. We examined two distinct scenarios concerning the use of titanium oxide. In Fig. 1a, we employed a titanium oxide volume fraction of 0.001. We observed that as we increased the Reynolds number from 100 to 1000, the average Nusselt number exhibited a corresponding increase. A rise in the Nusselt number signifies a favorable enhancement in the convection process, aligning with its inherent definition. In Fig. 1b, a volume fraction of 0.05 for titanium oxide was employed, and a similar trend in the Nusselt number concerning the Reynolds number was observed. This consistent pattern confirms the positive influence of increased convection, further affirming the results. In both cases, Fig. 3a,b plays a significant role in validating our simulation. Subsequently, we will delve into more in-depth post-processing within the results discussion.

Concluding our analysis, we now introduce Table 4, which provides a comprehensive overview of the absolute errors in the computation of energy Eq. (4). Within this table, we've meticulously assembled data from 20 parametric computations. Our aim was to pinpoint the minimum absolute error among these computations, and accordingly, we've arranged them in ascending order. This organization allows for a clear progression from the lowest to the highest absolute error values.

**Table 4:** Absolute error in computation of energy equation.

$\phi_{2}$	$\omega$	$\phi_{2}$	Re	$\left| {u_{1} \frac{\partial T}{{\partial x}} + u_{2} \frac{\partial T}{{\partial y}} = \alpha_{hnf} \left( {\frac{{\partial^{2} T}}{{\partial x^{2} }} + \frac{{\partial^{2} T}}{{\partial y^{2} }}} \right)} \right|$
0.001	0.01	0.01	400	3.15E-15
0.001	0.01	0.001	400	3.22E-15
0.001	0.01	0.04	400	4.25E-15
0.001	0.01	0.05	400	4.25E-15
0.04	0.01	0.001	100	5.18E-14
0.001	0.01	0.001	100	5.46E-14
0.01	0.01	0.001	100	5.53E-14
0.04	0.01	0.01	100	5.62E-14
0.001	0.01	0.01	100	5.66E-14
0.01	0.01	0.01	100	5.82E-14
0.04	0.01	0.04	100	6.45E-14
0.01	0.01	0.04	100	6.46E-14
0.04	0.01	0.05	100	6.52E-14
0.01	0.01	0.05	100	6.57E-14
0.05	0.01	0.001	100	8.09E-14
0.05	0.01	0.01	100	8.71E-14
0.001	0.01	0.04	100	8.92E-14
0.001	0.01	0.05	100	9.05E-14
0.05	0.01	0.05	100	9.41E-14
0.05	0.01	0.04	100	9.50E-14

## Results discussion

In this article, our endeavor has been to explore a photovoltaic thermal system using forced convection, employing two-dimensional Navier–Stokes and heat equations. To tackle the challenge of preventing the PV/T system from overheating, we've utilized a hybrid nanofluid coolant comprising titanium oxide and silver metal nanoparticles, expertly blended into water. Our PV/T configuration adheres to a straightforward design, with the topmost layer featuring polycrystalline silicon, a widely-used material in solar generator equipment. Immediately below, we've integrated an absorber crafted from copper. Completing the setup is a rectangular flow channel housing a rotating cylinder, strategically included to curtail cell temperature while bolstering electrical efficiency. We've harnessed the Reynolds number, rotation speed, and volume fraction of both nanoparticles as variables in this pursuit. Through our rigorous analysis, we've procured invaluable results pertaining to cell temperature and have subsequently assessed the cell efficiency.

### Expansion of temperature along the cell

**Figure 4:**
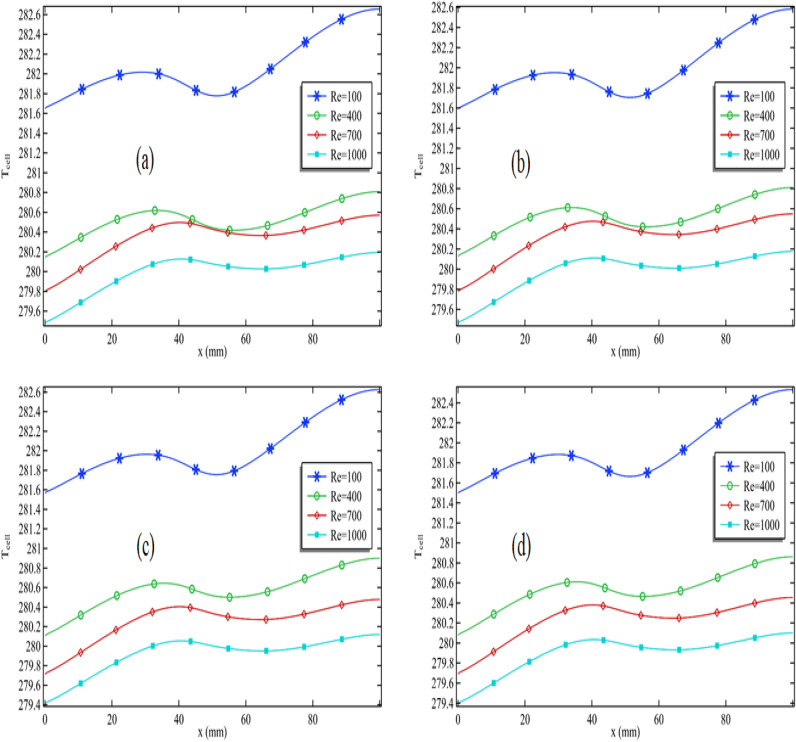
The cell temperature pattern for all Reynolds number when $\phi_{2}$ = 0.001 and $\omega$ = 0.01 when (**a**) $\phi_{1}$ = 0.001, (**b**) $\phi_{1}$ = 0.01, (**c**) $\phi_{1}$ = 0.04 and (**d**) $\phi_{1}$ = 0.05.

In Fig. 4a–d, we present the cell temperature as a function of length, maintaining constant values for the Reynolds number, volume fraction of silver, and cylinder rotation. To gain insights into the temperature pattern, we have varied the volume fraction of titanium oxide. In Fig. 4a, we observe that cell temperature increases along the length, albeit with fluctuations. Notably, as we reach the section where the cylinder is attached, the temperature starts to decline sharply. Furthermore, we find that increasing the Reynolds number from 100 to 400, and then from 400 to 700, leads to a significant decrease in cell temperature, indicating that a higher Reynolds number augments the convection process. Figure 4a also reveals a periodic temperature pattern, likely attributed to the movement of the cylinder. Examining Fig. 4a, the lowest cell temperature occurs at the initial length and increases towards the end. With rising Reynolds numbers, this minimum temperature diminishes. For instance, when the Reynolds number ranges from 100 to 400 in Fig. 4a, the minimum temperature decreases by 0.5%. Overall, within the Reynolds number range of 100 to 1000, the minimum temperature sees a reduction of 0.7%.

Moving on to Fig. 4b–d, we have increased the volume fraction of titanium oxide while keeping other parameters constant. A clear trend emerges, where the temperature is at a minimum at the beginning of the cell and reaches its peak towards the length's end. Augmenting the quantity of titanium oxide leads to a shift in both the minimum and maximum temperatures towards the lower end. Although the effect is not dramatic, it is discernible in Fig. 4b–d.

For instance, in Fig. 4a, with a Reynolds number of 1000, the minimum temperature at the initial length is approximately 279.6 K. In Fig. 4d, we observe a similar scenario, with the minimum temperature at the initial length measuring around 279.4 K. While this change isn't substantial, some improvement is evident. The increase in the Reynolds number accelerates fluid speed, amplifying the convection process. Notably, nanofluids are favored coolants due to their heightened thermal conductivity, rendering them advantageous in this context.

**Figure 5:**
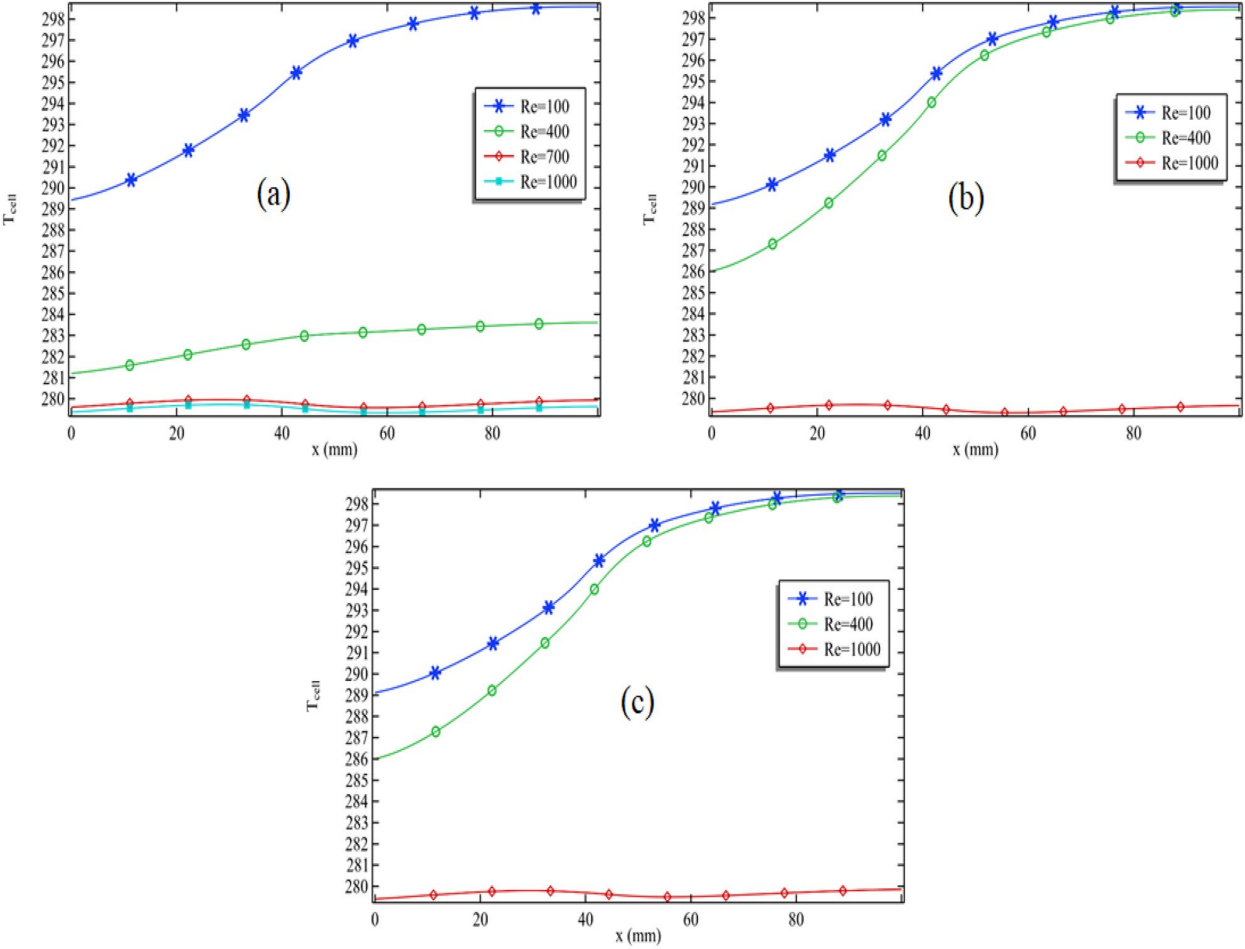
The cell temperature pattern for all Reynolds number when $\phi_{2}$ = 0.001 and $\phi_{1}$ = 0.001 when (**a**) $\omega$ = 0.05 m/s, (**b**) $\omega$ = 0.09 m/s and (**c**) $\omega$ = 0.1 m/s.

In Fig. 5a–c, we've presented a series of plots showcasing the cell temperature in relation to its length. However, in these cases, we've kept the proportions of both nanoparticle types constant while varying the speed of the circular cylinder from 0.05 to 0.1 m/s. The primary objective of these figures is to investigate whether an increase in the cylinder's rotational speed leads to a reduction in cell temperature. Upon examining Fig. 5a, where the cylinder's speed is set at 0.01 m/s, we can observe a consistent decrease in cell temperature as the nanofluid passes through the channel. However, an intriguing phenomenon comes to light in Fig. 5a. As the cylinder's speed reaches 0.05 m/s, there is a sudden temperature increase. This observation suggests that augmenting the cylinder's rotation speed doesn't necessarily contribute to a reduction in cell temperature.

Contrastingly, in Fig. 5b,c, we find that the Reynolds number still plays a pivotal role in reducing cell temperature. This discrepancy raises a noteworthy question: why does an increase in cylinder rotation fail to achieve the anticipated cooling effect? To provide further context, let's consider Figs. 4a and 5a when Re = 100. In Fig. 4a, the minimum temperature at the initial length measures 281.6 K. However, in Fig. 5a with the same Reynolds number, it's notably higher at 289.5 K. This marked difference implies that increasing the cylinder's rotation speed doesn't align with the expected reduction in temperature. However, it's essential to recognize that despite this unexpected behavior, a significant amount of thermal energy has been gained through this process. Such surplus thermal energy might well find valuable applications in various contexts. In conclusion, when we compare Fig. 5b,c at Re = 100, we continue to observe slight fluctuations in the minimum temperature at the initial length, suggesting that the cylinder's rotation is displaying rather intriguing characteristics.

**Figure 6:**
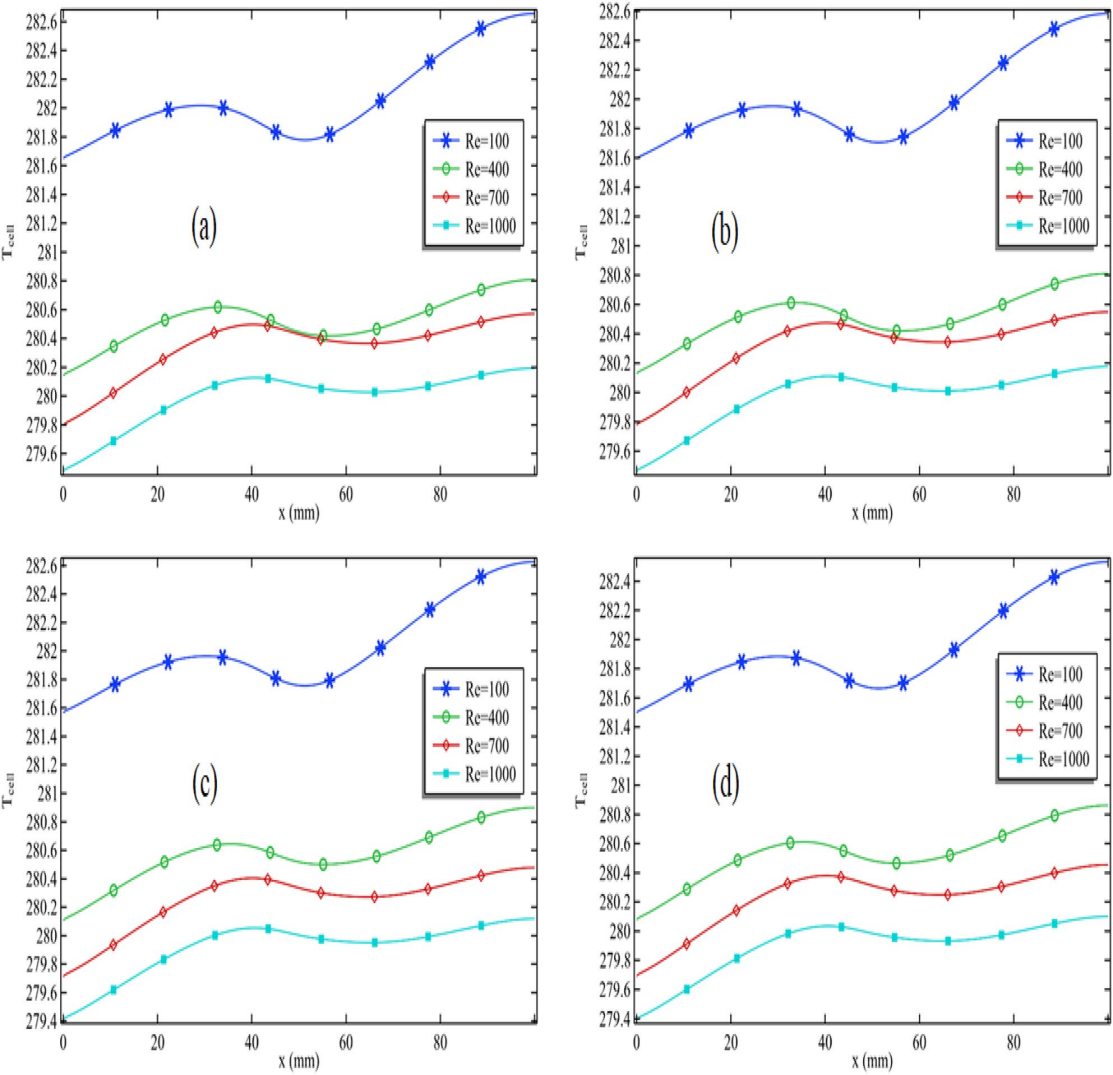
The cell temperature pattern for all Reynolds number when $\phi_{1}$ = 0.001 and $\omega$ = 0.01 when (**a**) $\phi_{2}$ = 0.001, (**b**) $\phi_{2}$ = 0.01, (**c**) $\phi_{2}$ = 0.04 and (**d**) $\phi_{2}$ = 0.05.

In Fig. 6a–d, we maintained the volume fraction of titanium oxide on a circular cylinder as a constant, aiming to examine how cell temperature behaves when altering the volume fraction of silver. Upon examining all the Fig. 6a–d presented in Fig. 6, a distinct trend emerges when Re = 1000 and the volume fraction of silver is varied from 0.001 to 0.05. In Fig. 6a, a minimum temperature of 279.5 K is observed, while in Fig. 6d, a slightly lower minimum temperature of 279.4 K is noted, representing a minor reduction of only 0.1 K. While this temperature decrease isn't substantial, it signifies a noticeable drop in the minimum temperature.

Additionally, it's worth noting that when Re = 100, there's a clear temperature decrease in the region where the cylinder is attached. However, when the Reynolds number surpasses 100, a shift occurs, and the temperature in that area begins to increase. This impact is distinctly visible in Fig. 4a–d. Hence, it can be reasonably inferred that when maintaining a lower Reynolds number, the rotation of the cylinder proves to be advantageous.

### Pattern and enhancement in cell efficiency

The formula for cell efficiency is provided in (9). We designed this simulation to analyze a system's capability to generate energy through solar radiation, using specific parameters. This section was developed to investigate the parameters contributing to increased cell efficiency.

**Figure 7:**
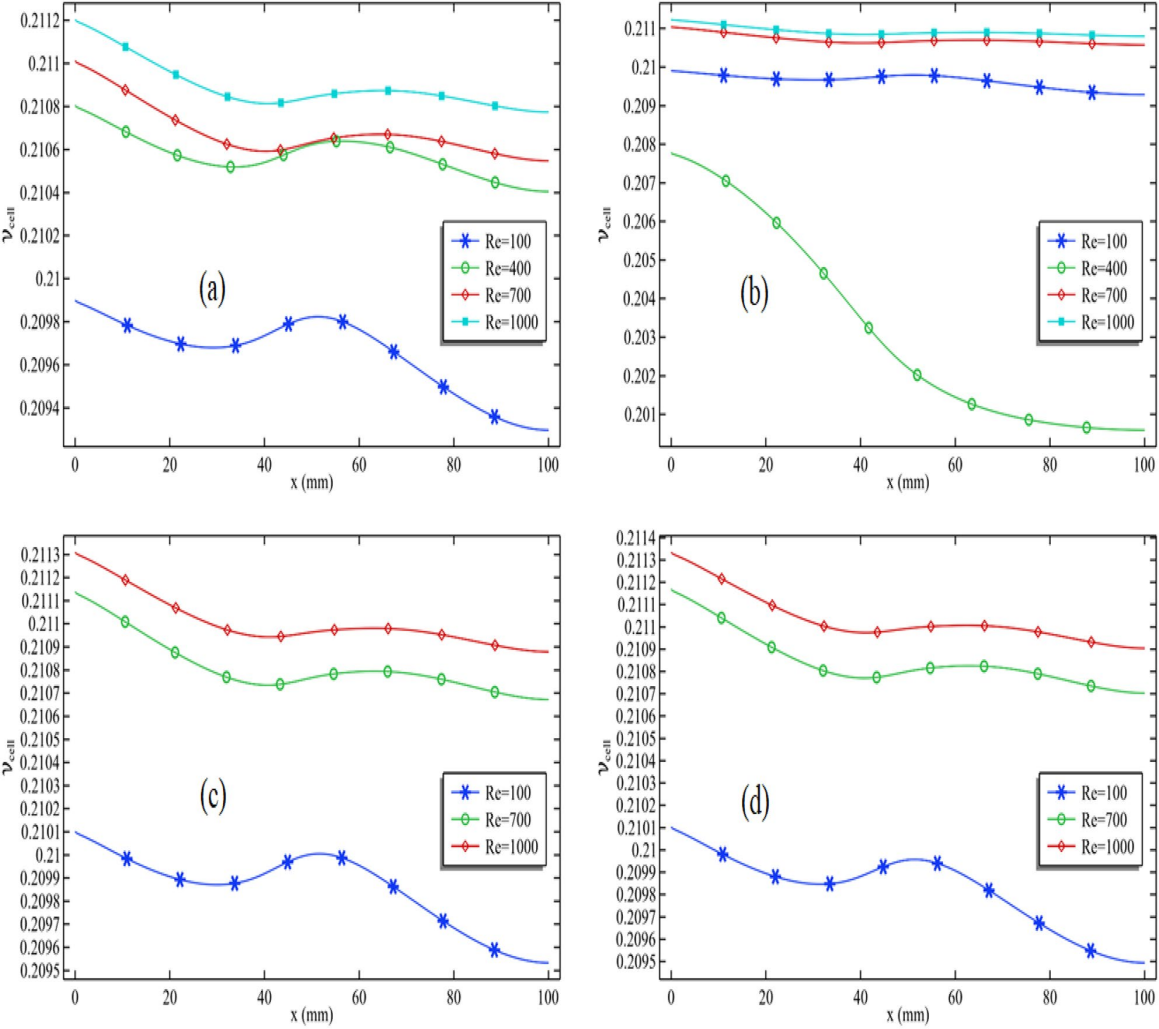
Cell efficiency for all Reynolds number when $\phi_{1}$ = 0.001 and $\omega$ = 0.01 when (**a**) $\phi_{2}$ = 0.001, (**b**) $\phi_{2}$ = 0.01 and (**c**) $\phi_{2}$ = 0.04 and (**d**) $\phi_{2}$ = 0.05.

In Fig. 7a–d, we assessed cell efficiency across a particular graph length while keeping the Reynolds number constant. These figures display the variation in silver nanoparticle concentration and Reynolds number. Figure 7a illustrates that cell efficiency generally decreases along the length, with varying impacts when the circular cylinder is attached and in motion at a specific speed. Notably, in Fig. 7a, when the Reynolds number is moderate (Re = 100), cell efficiency experiences an increase in that area. However, under different conditions, cell efficiency decreases in the region where the cylinder is attached, particularly with higher Reynolds numbers (Re > 400). The maximum cell efficiency is achieved at the length's beginning, and as the Reynolds number rises, cell efficiency at this initial point also improves. Consequently, the overall cell efficiency substantially increases with an escalating Reynolds number.

As observed in Fig. 7d, when the Reynolds number is 100, the maximum cell efficiency registers at 0.2101. When the Reynolds number increases to Re = 1000, the cell efficiency rises slightly to 0.2113. This means that elevating the Reynolds number from 100 to 1000 results in a 0.57% increase in cell efficiency. In summary, analyzing all the figures in Fig. 4a–d, it becomes evident that the addition of nanoparticles significantly enhances the performance of the PV/T system.

**Figure 8:**
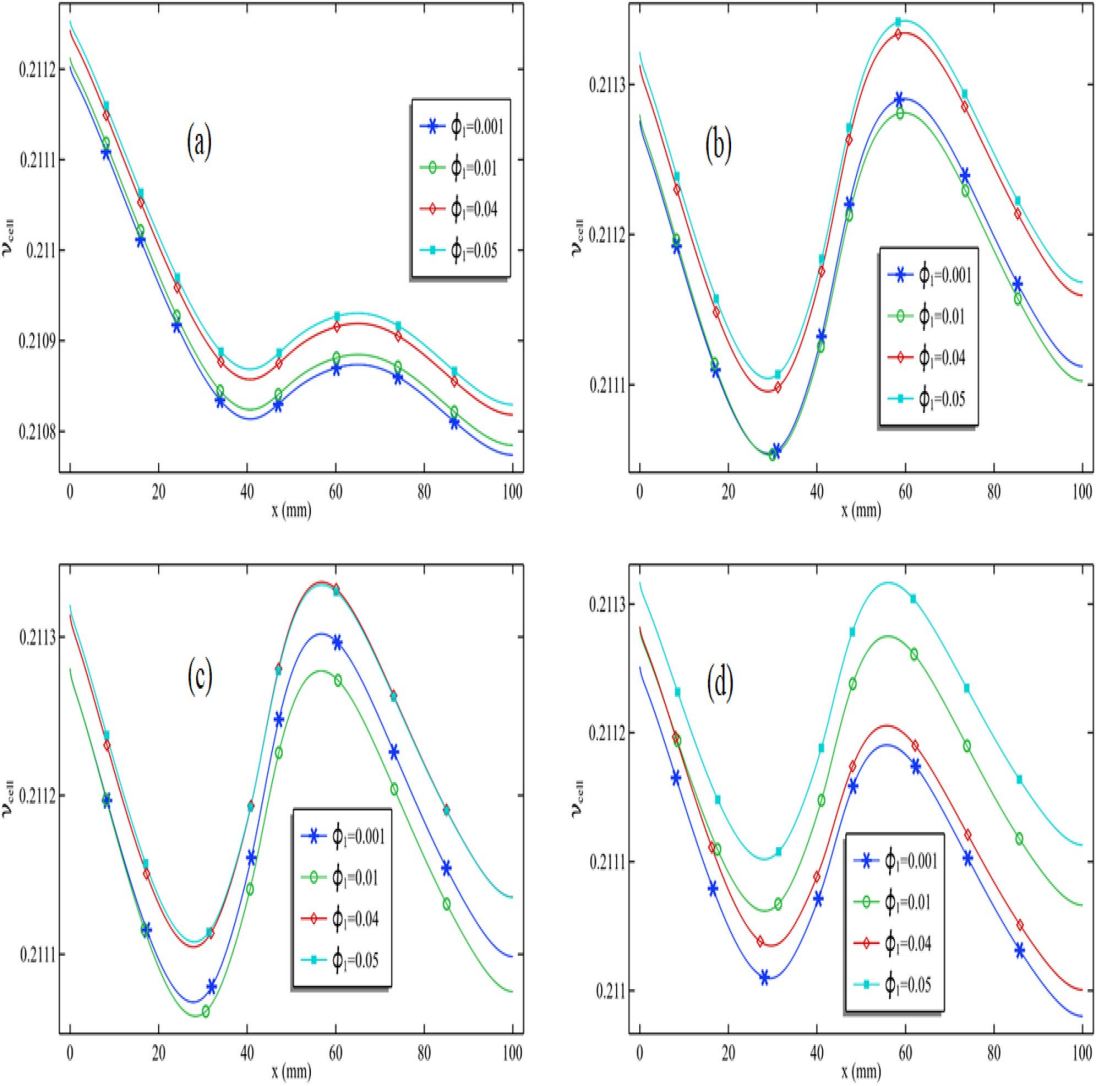
Cell efficiency for all volume fraction of titanium oxide when $\phi_{2}$ = 0.001 and Re = 1000 when (**a**) $\omega$ = 0.01, (**b**) $\omega$ = 0.05 and (**c**) $\omega$ = 0.09 and (**d**) $\omega$ = 0.1

In Fig. 8a–d, we maintained the Reynolds number at 1000 and the concentration of silver nanoparticles at 0.001. We conducted an analysis of cell efficiency while introducing variations in the volume fraction of titanium oxide and the rotation speed of the cylinder. Figure 8a demonstrates that for a constant volume fraction of titanium oxide, cell efficiency tends to decrease. Notably, in this configuration Fig. 8a, with the cylinder rotating at 0.01 m/s, the cell efficiency experiences a positive influence from the cylinder's rotation speed. Moreover, a recurring pattern in cell efficiency along the length can be observed, similar to what was seen in Fig. 7a. Furthermore, in Fig. 8a, increasing the volume fraction of titanium oxide leads to a substantial improvement in cell efficiency, with the maximum efficiency observed at the initial cell length.

Next, in Fig. 8b, we maintained the rotation speed of the cylinder at 0.05 m/s. Notably, as we reduce the volume fraction of titanium oxide from 0.01 to 0.001, there is a decrease in cell efficiency, including the average cell efficiency. However, upon increasing the volume fraction of titanium oxide from 0.01 to 0.05, the average cell efficiency experiences an increase. By synthesizing the observations from Fig. 8c,d, we can deduce that using a 4% or 5% volume fraction of titanium oxide is more effective when the cylinder operates at higher speeds.

**Figure 9:**
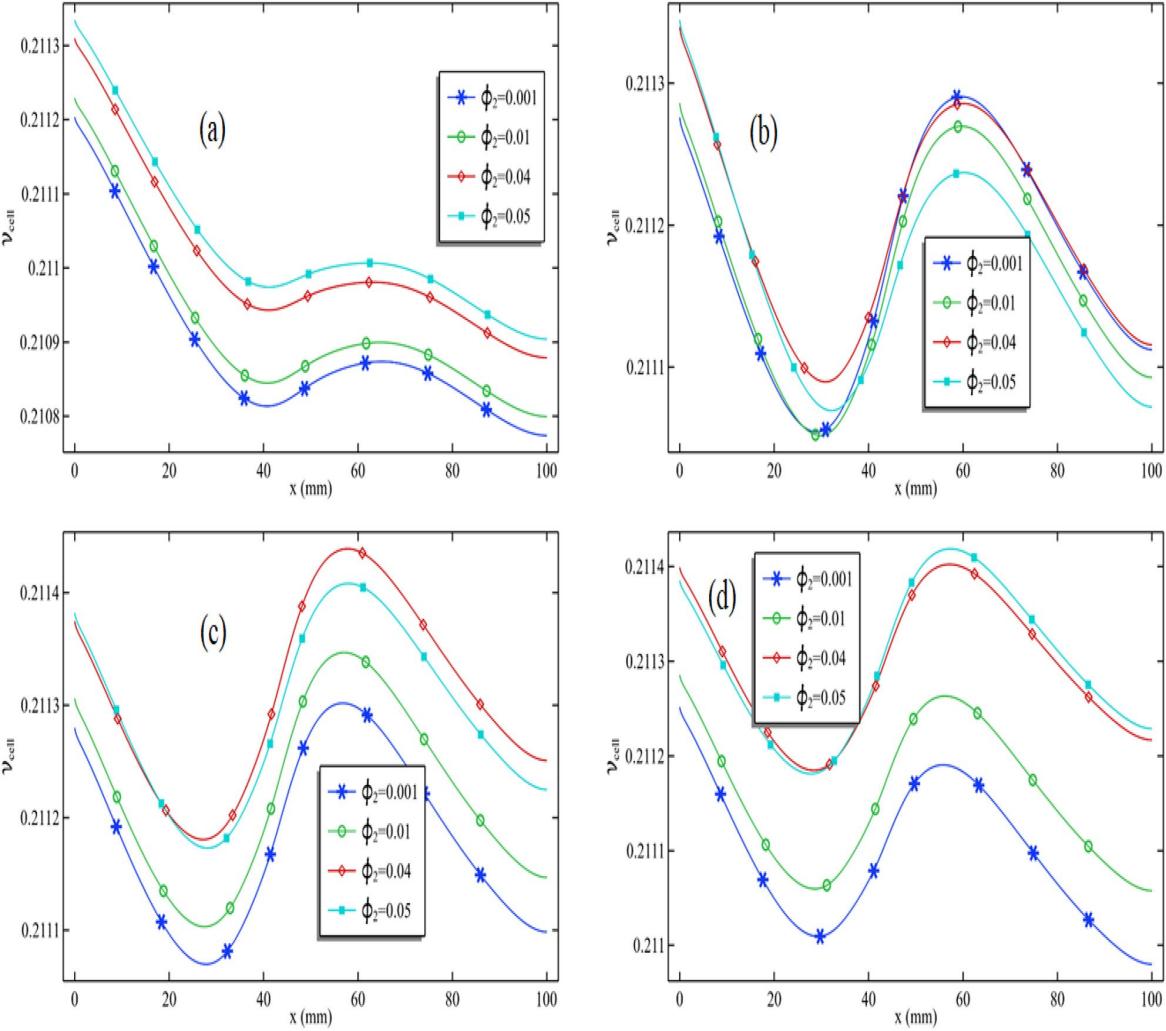
Cell efficiency for all volume fraction of silver when $\phi_{1}$ = 0.001 and Re = 1000 when (**a**) $\omega$ = 0.01, (**b**) $\omega$ = 0.05 and (**c**) $\omega$ = 0.09 and (**d**) $\omega$ = 0.1

We generated Fig. 9a–d to explore the impact of silver nanoparticles and their role in enhancing cell efficiency. A comparison between Figs. 8a and 9a confirms that silver nanoparticles have a substantially more pronounced effect in improving cell efficiency in contrast to titanium oxide. In Fig. 9a, it is evident that an increase in the volume fraction of silver nanoparticles results in a remarkable enhancement of cell efficiency. This graph was generated with the cylinder operating at 0.01 m/s. In Fig. 9b, distinct patterns emerge when the cylinder's rotation speed is set at 0.05 m/s. Here, it can be observed that cell efficiency experiences an initial increase, followed by a decline as the volume fraction of silver nanoparticles increases.

Both Fig. 9c,d exhibit a recurring pattern in cell efficiency. In Fig. 9c, the maximum cell efficiency is achieved when the volume fraction of silver is 4% and the cylinder operates at 0.09 m/s. In Fig. 9d, the maximum cell efficiency is reached when the volume fraction of silver is 0.05, and the cylinder speed is 0.1 m/s. This indicates that we can regulate cell efficiency by manipulating the Reynolds number, adjusting the volume fractions of silver and titanium oxide, and controlling the cylinder speed.

Finally, we are presenting Table 5 in which we calculated the total volume fraction of nanoparticles and the maximum cell efficiency. In this table, we fixed the volume fraction of silver nanoparticles at 5%. The table highlights that when the cylinder speed is set at 0.9 m/s, Re = 1000, and $\phi_{1}$ = $\phi_{2}$ = 0.05, maximum cell efficiency can be achieved.

**Table 5:** Them maximum cell efficiency when volume fraction of silver $\phi_{2}$ = 0.05.

$\phi_{2}$	$\omega$	$\phi_{2}$	Re	$\phi$	$\nu_{cell}$
0.05	0.01	0.001	100	0.051	0.210102
0.05	0.01	0.001	700	0.051	0.211169
0.05	0.01	0.001	1000	0.051	0.211334
0.05	0.01	0.01	100	0.06	0.210113
0.05	0.01	0.01	700	0.06	0.211179
0.05	0.01	0.01	1000	0.06	0.211342
0.05	0.01	0.04	100	0.09	0.210179
0.05	0.01	0.04	700	0.09	0.211214
0.05	0.01	0.04	1000	0.09	0.21137
0.05	0.01	0.05	100	0.1	0.21021
0.05	0.01	0.05	700	0.1	0.211227
0.05	0.01	0.05	1000	0.1	0.21138
0.05	0.05	0.001	100	0.051	0.20572
0.05	0.05	0.001	400	0.051	0.210907
0.05	0.05	0.001	700	0.051	0.211236
0.05	0.05	0.001	1000	0.051	0.211344
0.05	0.05	0.01	100	0.06	0.205758
0.05	0.05	0.01	400	0.06	0.210969
0.05	0.05	0.01	700	0.06	0.211249
0.05	0.05	0.01	1000	0.06	0.211352
0.05	0.05	0.04	100	0.09	0.205892
0.05	0.05	0.04	400	0.09	0.210973
0.05	0.05	0.04	700	0.09	0.211284
0.05	0.05	0.04	1000	0.09	0.2114
0.05	0.05	0.05	100	0.1	0.205939
0.05	0.05	0.05	400	0.1	0.210982
0.05	0.05	0.05	700	0.1	0.211295
0.05	0.05	0.05	1000	0.1	0.211409
0.05	0.09	0.001	100	0.051	0.20584
0.05	0.09	0.001	400	0.051	0.207806
0.05	0.09	0.001	700	0.051	0.211213
0.05	0.09	0.001	1000	0.051	0.211408
0.05	0.09	0.01	100	0.06	0.205878
0.05	0.09	0.01	400	0.06	0.20784
0.05	0.09	0.01	700	0.06	0.211204
0.05	0.09	0.01	1000	0.06	0.211413
0.05	0.09	0.04	100	0.09	0.206011
0.05	0.09	0.04	400	0.09	0.20797
0.05	0.09	0.04	700	0.09	0.211247
0.05	0.09	0.04	1000	0.09	0.211467
0.05	0.09	0.05	100	0.1	0.206057
0.05	0.09	0.05	400	0.1	0.208011
0.05	0.09	0.05	700	0.1	0.211253
0.05	0.09	0.05	1000	0.1	0.211472
0.05	0.1	0.001	100	0.051	0.205869
0.05	0.1	0.001	400	0.051	0.207761
0.05	0.1	0.001	700	0.051	0.211189
0.05	0.1	0.001	1000	0.051	0.211419
0.05	0.1	0.01	100	0.06	0.205908
0.05	0.1	0.01	400	0.06	0.207797
0.05	0.1	0.01	1000	0.06	0.211409
0.05	0.1	0.04	100	0.09	0.206041
0.05	0.1	0.04	400	0.09	0.207923
0.05	0.1	0.04	700	0.09	0.21126
0.05	0.1	0.04	1000	0.09	0.211463
0.05	0.1	0.05	100	0.1	0.206086
0.05	0.1	0.05	400	0.1	0.207967
0.05	0.1	0.05	700	0.1	0.211251
0.05	0.1	0.05	1000	0.1	0.211462

## Conclusion

In this comprehensive research endeavor, we embarked on a thorough investigation of a photovoltaic thermal (PV/T) system with the primary objective of significantly enhancing its electrical production capabilities. Our approach centered on the utilization of a water-based nanofluid, a remarkable hybrid blend comprising titanium oxide and silver nanoparticles. The PV/T system itself boasted an elegantly straightforward configuration, consisting of three distinct layers. At the pinnacle lay a layer of polycrystalline silicon, followed by a copper absorber nestled in the middle, all enveloped within a flow channel designed for efficient cooling. A standout feature of this PV/T system was the strategic placement of a rotating circular cylinder within the central region of the flow channel, operating at a specific speed. Our investigative efforts spanned a range of critical parameters, including the Reynolds number, volume fractions of both nanoparticles, and the cylinder's rotational speed, yielding the following noteworthy findings:

- The cell temperature increases along the length, yet the increment is not consistent throughout. This temperature pattern exhibits periodicity, possibly influenced by the cylinder's movement.
- Increasing the Reynolds number enhances the convection process and significantly reduces cell temperature. A higher Reynolds number elevates fluid speed, thereby improving convection.
- Increasing the rotation speed of the cylinder does not effectively reduce temperature. However, it results in a notable increase in thermal energy, which may have practical applications.
- Varying the volume fraction of silver from 0.001 to 0.05 leads to a reduction in the minimum temperature along the length.
- Increasing the Reynolds number boosts cell efficiency at the length's initial portion and substantially improves the average cell efficiency.
- The concentration of silver nanoparticles has both positive and negative impacts on cell efficiency, with the highest efficiency occurring at the length's beginning.
- The inclusion of titanium oxide substantially improves cell efficiency, with the maximum efficiency observed at the length's beginning.
- Utilizing a 4% or 5% volume fraction of titanium oxide is preferable when the cylinder speed is high.
- Silver nanoparticles prove more effective in increasing cell efficiency compared to titanium oxide.
- A higher volume fraction of silver nanoparticles significantly enhances cell efficiency, although the efficiency pattern changes with cylinder speed.
- Cell efficiency can be controlled by adjusting the Reynolds number, volume fraction of silver and titanium oxide, and cylinder speed in combination.

### Future scope

In this comprehensive article, we explore into the particulars of a photovoltaic thermal system, a system thoughtfully designed with a central rotating cylinder within the channel. Our primary goal was to tackle the challenge of reducing the system's temperature to enhance its electrical efficiency. This endeavor holds the potential to evolve into a broader exploration that incorporates multiple cylinders strategically positioned at various intervals along the channel's length. Within this expanded framework, the use of materials with exceptional thermal conductivity for these cylinders becomes crucial, offering an effective solution to combat rising temperatures caused by solar radiation.

Additionally, we can explore the application of alternative nanofluid models, such as tri-nanofluids, to further amplify the system's electrical efficiency. The significance of this research extends beyond the mere construction of a photovoltaic thermal system. It encompasses the fundamental principle of maintaining a consistent increase in fluid velocity as it enters the system. This crucial element is vital in optimizing the overall system performance and underscores the core focus of our study.

## Data Availability

All data used in this manuscript have been presented within the manuscript. No data are hidden or restricted.

## References

[1] Good C, Andresen I, Hestnes AG (2015). Solar energy for net zero energy buildings—A comparison between solar thermal, PV and photovoltaic–thermal (PV/T) systems. Solar Energy.

[2] Al-Waeli AH, Sopian K, Kazem HA, Chaichan MT (2017). Photovoltaic/Thermal (PV/T) systems: Status and future prospects. Renew. Sustain. Energy Rev..

[3] Han, Z. *Nanofluids with enhanced thermal transport properties* (Doctoral dissertation) (2008).

[4] Michaelides EE (2013). Transport properties of nanofluids. A critical review. J. Non-Equilib. Thermodyn..

[5] Tiwari A, Sodha MS (2006). Performance evaluation of hybrid PV/thermal water/air heating system: A parametric study. Renew. Energy.

[6] Tiwari A, Dubey S, Sandhu GS, Sodha MS, Anwar SI (2009). Exergy analysis of integrated photovoltaic thermal solar water heater under constant flow rate and constant collection temperature modes. Appl. Energy.

[7] Tonui JK, Tripanagnostopoulos Y (2007). Improved PV/T solar collectors with heat extraction by forced or natural air circulation. Renew. Energy.

[8] Barone G, Buonomano A, Forzano C, Palombo A, Panagopoulos O (2019). Photovoltaic thermal collectors: Experimental analysis and simulation model of an innovative low-cost water-based prototype. Energy.

[9] Bianco V, Scarpa F, Tagliafico LA (2018). Numerical analysis of the Al_2_O_3_-water nanofluid forced laminar convection in an asymmetric heated channel for application in flat plate PV/T collector. Renew. Energy.

[10] Chow TT, Pei G, Fong KF, Lin Z, Chan ALS, Ji J (2009). Energy and exergy analysis of photovoltaic–thermal collector with and without glass cover. Appl. Energy.

[11] Ebrahimnia-Bajestan E, Moghadam MC, Niazmand H, Daungthongsuk W, Wongwises S (2016). Experimental and numerical investigation of nanofluids heat transfer characteristics for application in solar heat exchangers. Int. J. Heat Mass Transf..

[12] Gorji TB, Ranjbar AA (2017). Thermal and exergy optimization of a nanofluid-based direct absorption solar collector. Renew. Energy.

[13] Herrando M, Ramos A, Zabalza I, Markides CN (2019). A comprehensive assessment of alternative absorber-exchanger designs for hybrid PVT-water collectors. Appl. Energy.

[14] Jia Y, Alva G, Fang G (2019). Development and applications of photovoltaic–thermal systems: A review. Renew. Sustain. Energy Rev..

[15] Jia Y, Ran F, Zhu C, Fang G (2020). Numerical analysis of photovoltaic-thermal collector using nanofluid as a coolant. Solar Energy.

[16] Mahian O, Kianifar A, Sahin AZ, Wongwises S (2014). Entropy generation during Al_2_O_3_/water nanofluid flow in a solar collector: Effects of tube roughness, nanoparticle size, and different thermophysical models. Int. J. Heat Mass Transf..

[17] Nahar A, Hasanuzzaman M, Rahim NA (2017). Numerical and experimental investigation on the performance of a photovoltaic thermal collector with parallel plate flow channel under different operating conditions in Malaysia. Solar Energy.

[18] Nasrin R, Rahim NA, Fayaz H, Hasanuzzaman M (2018). Water/MWCNT nanofluid based cooling system of PVT: Experimental and numerical research. Renew. Energy.

[19] Pang W, Cui Y, Zhang Q, Wilson GJ, Yan H (2020). A comparative analysis on performances of flat plate photovoltaic/thermal collectors in view of operating media, structural designs, and climate conditions. Renew. Sustain. Energy Rev..

[20] Prakash J (1994). Transient analysis of a photovoltaic-thermal solar collector for co-generation of electricity and hot air/water. Energy Convers. Manage..

[21] Memon AA, Memon MA, Haque MM (2023). Numerical investigation of electrical efficiency with the application of hybrid nanofluids for photovoltaic thermal systems contained in a cavity channel. J. Math..

[22] Memon AA, Khan WA, Muhammad T (2023). Numerical investigation of photovoltaic thermal energy efficiency improvement using the backward step containing Cu–Al_2_O_3_ hybrid nanofluid. Alex. Eng. J..

[23] Alghamdi M, Memon AA, Muhammad T, Ali MR (2023). A numerical investigation of a photovoltaic thermal system contained a trapezoidal channel with transport of silver and titanium oxide using the water as base fluids. Case Stud. Therm. Eng..

[24] Aglawe, K.R., Yadav, R.K., & Thool, S.B. Fabrication, experimentation and numerical simulation of micro channel heat sink for enhancing thermal performance of electronic devices. *Int. J. Interact. Des. Manuf. (IJIDeM)*, pp.1–16 (2023).

[25] Yousefi E, Nejad AA, Sayyar N (2023). A new approach for simultaneous thermal management of hot and cold sides of thermoelectric modules. Appl. Therm. Eng..

[26] Karaaslan I, Menlik T (2021). Numerical study of a photovoltaic thermal (PV/T) system using mono and hybrid nanofluid. Solar Energy.

[27] Elhag SH, Memon AA, Memon MA, Bhatti K, Jacob K, Alzahrani S, Seidu J (2022). Analysis of forced convection with hybrid Cu–Al_2_O_3_ nanofluids injected in a three-dimensional rectangular channel containing three perpendicular rotating blocks with turbulent modeling. J. Nanomater..

[28] Pak BC, Cho YI (1998). Hydrodynamic and heat transfer study of dispersed fluids with submicron metallic oxide particles. Exp. Heat Transf. Int. J..

